# Safety and efficacy of mTOR inhibitor treatment in patients with tuberous sclerosis complex under 2 years of age – a multicenter retrospective study

**DOI:** 10.1186/s13023-019-1077-6

**Published:** 2019-05-03

**Authors:** Afshin Saffari, Ines Brösse, Adelheid Wiemer-Kruel, Bernd Wilken, Paula Kreuzaler, Andreas Hahn, Matthias K. Bernhard, Cornelis M. van Tilburg, Georg F. Hoffmann, Matthias Gorenflo, Sven Hethey, Olaf Kaiser, Stefan Kölker, Robert Wagner, Olaf Witt, Andreas Merkenschlager, Andreas Möckel, Timo Roser, Jan-Ulrich Schlump, Antje Serfling, Juliane Spiegler, Till Milde, Andreas Ziegler, Steffen Syrbe

**Affiliations:** 1 Division of Child Neurology and Metabolic Medicine, Center for Paediatrics and Adolescent Medicine, University Hospital Heidelberg, Heidelberg, Germany; 2 Clinic for Children and Adolescents, Epilepsy Centre Kork, Kehl-Kork, Germany; 3 Department of Pediatric Neurology, Kassel Hospital, Kassel, Germany; 4 Department of Child Neurology, University Hospital, Gießen, Germany; 5 Department of Neuropediatrics, University Hospital of Children, Leipzig, Germany; 6 Hopp Children’s Cancer Center Heidelberg (KiTZ), Heidelberg, Germany; 7 Clinical Cooperation Unit Pediatric Oncology, German Cancer Research Center (DKFZ) and German Consortium for Translational Cancer Research (DKTK), Heidelberg, Germany; 8 KiTZ Clinical Trial Unit (ZIPO), Department of Pediatric Hematology and Oncology, Heidelberg University Hospital, Heidelberg, Germany; 9 Department for Congenital Heart Defects/Paediatric Cardiology, Heidelberg University Hospital, Heidelberg, Germany; 10 Auf der Bult – Center for Children and Adolescents, Hannover, Germany; 11 Department of Paediatrics I, Paediatric Neurology, University Hospital Essen, University of Duisburg-Essen, Essen, Germany; 12 Department of Pediatric Cardiology, University of Leipzig, Heart Center, Leipzig, Germany; 13 Sana Kliniken Leipziger Land, Borna, Germany; 14 Department of Paediatric Neurology and Developmental Medicine, Hauner Children’s Hospital, University of Munich, Munich, Germany; 15 Division for Children and Adolescents, Evangelical Hospital Oberhausen, Oberhausen, Germany; 16 Department of Pediatrics, University Medical Center Schleswig-Holstein, Campus Lübeck, Germany

**Keywords:** Tuberous sclerosis complex, mTOR inhibitor, Everolimus, Children, Neonates

## Abstract

**Background:**

Tuberous sclerosis complex (TSC) is a multisystem disease with prominent neurologic manifestations such as epilepsy, cognitive impairment and autism spectrum disorder. mTOR inhibitors have successfully been used to treat TSC-related manifestations in older children and adults. However, data on their safety and efficacy in infants and young children are scarce. The objective of this study is to assess the utility and safety of mTOR inhibitor treatment in TSC patients under the age of 2 years.

**Results:**

A total of 17 children (median age at study inclusion 2.4 years, range 0–6; 12 males, 5 females) with TSC who received early mTOR inhibitor therapy were studied. mTOR inhibitor treatment was started at a median age of 5 months (range 0–19 months). Reasons for initiation of treatment were cardiac rhabdomyomas (6 cases), subependymal giant cell astrocytomas (SEGA, 5 cases), combination of cardiac rhabdomyomas and SEGA (1 case), refractory epilepsy (4 cases) and disabling congenital focal lymphedema (1 case). In all cases everolimus was used. Everolimus therapy was overall well tolerated. Adverse events were classified according to the *Common Terminology Criteria of Adverse Events* (CTCAE, Version 5.0). Grade 1–2 adverse events occurred in 12 patients and included mild transient stomatitis (2 cases), worsening of infantile acne (1 case), increases of serum cholesterol and triglycerides (4 cases), changes in serum phosphate levels (2 cases), increase of cholinesterase (2 cases), transient neutropenia (2 cases), transient anemia (1 case), transient lymphopenia (1 case) and recurrent infections (7 cases). No grade 3–4 adverse events were reported. Treatment is currently continued in 13/17 patients. Benefits were reported in 14/17 patients and included decrease of cardiac rhabdomyoma size and improvement of arrhythmia, decrease of SEGA size, reduction of seizure frequency and regression of congenital focal lymphedema. Despite everolimus therapy, two patients treated for intractable epilepsy are still experiencing seizures and another one treated for SEGA showed no volume reduction.

**Conclusion:**

This retrospective multicenter study demonstrates that mTOR inhibitor treatment with everolimus is safe in TSC patients under the age of 2 years and shows beneficial effects on cardiac manifestations, SEGA size and early epilepsy.

## Background

Tuberous sclerosis complex (TSC) is an autosomal dominant neurodevelopmental disorder caused by loss-of-function mutations in the *TSC1* and *TSC2* genes, encoding the Tuberin-Hamartin complex, acting as a critical upstream suppressor of the mammalian target of rapamycin (mTOR), a key signaling pathway controlling cellular growth and metabolism. TSC is a multisystem disease, and about 90% of individuals develop central nervous system complications, such as epilepsy, cognitive impairment and autism spectrum disorder [1, 2]. Epilepsy, usually starting during the first 3 years of life, occurs in 83.5% of affected patients and represents the most prevalent and challenging clinical manifestation of TSC [2, 3]. Early-onset epilepsy typically presents with focal seizures with or without secondary generalization often evolving into infantile spasms (IS) during infancy. IS or West syndrome, a severe epileptic encephalopathy characterized by epileptic spasms and a pathognomonic EEG pattern (hypsarrhythmia), occurs in 38.8% of TSC patients [3]. Antiepileptic treatment with vigabatrin or adrenocortictropic hormone (ACTH) has proven to stop IS in 71.5% of TSC patients [3].

mTOR inhibition is a promising molecular target for the treatment of TSC manifestations, including epilepsy and behavior. The mTOR inhibitors rapamycin (sirolimus) and everolimus have been increasingly used in TSC. Everolimus has gained approval for the treatment of TSC-related subependymal giant cell astrocytomas (SEGA) and renal angiomyolipomas. In a phase I/II clinical trial including 20 children with TSC-related refractory epilepsy above the age of 2 years, everolimus led to a median seizure reduction of 73% with only mild to moderate adverse effects [4]. Recently, the EXIST-3 trial showed a significant seizure reduction in a large cohort of TSC patients aged 2–65 years treated with everolimus [5] and led to the additional approval for the treatment of refractory focal seizures in TSC above 2 years of age.

TSC-related early-onset epilepsy in the first year of life and in particular infantile spasms (IS) are the most important risk factors for mental impairment and autism spectrum disorder. In TSC, 74.4% of patients with IS develop cognitive impairment, while only 39.2% of patients without IS show neurologic deficits [6, 7]. It can be hypothesized that early initiation of mTOR inhibitor treatment reduces the risk for TSC-related neuropsychiatric deficits [8–10]. However, data on safety and efficacy of everolimus treatment in infants and young children are still lacking and only one study analyzed clinical data of TSC patients treated with everolimus within the first 2 years of life [11].

The main purpose of this study is to collect data on the tolerability of mTOR inhibitors and the occurrence of adverse events in neonates and infants under 2 years of age. We further aimed at collecting preliminary data on the effects of mTOR inhibitor treatment on TSC-related malformations, epilepsy and neurodevelopment.

## Results

### Cohort

We collected data from 17 patients with a definite (clinical and/or genetic) diagnosis of TSC according to the *2012 International TSC Consensus Conference* [12] (for clinical data see Table 1). Twelve patients were male, 5 female. Median age at inclusion into this study was 2.4 years (range 0–6 years). The median period of follow-up was 2.1 years (range 0.5–5.5 years). Diagnosis of TSC was suspected prenatally due to cardiac rhabdomyomas in 14 cases and due to a SEGA in 1 case (patient #12). Two patients initially presented with focal seizures and neuroimaging was suggestive of TSC (patients #1 and #16). In addition, we collected follow-up data in one case (patient #14) that has been published previously [13].

**Table 1:** Clinical data

Patient #	Sex	Age at inclusion [y]	Age at Diagnosis [d]	Mutation	sporadic / familial	First clinical manifestation	Reason for mTOR inhibition	Further manifestations
1	f	4.3	210	*TSC2*	sporadic	Epilepsy	SEGA	Neuro: tubers, SEN, migration lines, DD
2	m	6.2	prenatal	*TSC2*	sporadic	CR	Congenital focal lymphedema	Skin: subungual fibromas Neuro: Epilepsy, tubers, SEN, DD Other: lumbar scoliosis, aneurysms, renal artery stenosis
3	f	0	prenatal	NA	familial	CR	SEGA	Skin: angiofibromas, white spots, Shagreen patch Neuro: Epilepsy, tubers, DD
4	m	0.1	49	NA	sporadic	CR	SEGA	Skin: angiofibromas, white spots Neuro: Epilepsy, tubers, DD
5	m	1.5	60	NA	sporadic	CR	Epilepsy	Skin: white spots Neuro: tubers, SEN, DD Other: retinal hamartomas
6	m	0	prenatal	*TSC2*	familial	CR	CR	None
7	f	3.1	prenatal	*TSC2*	sporadic	CR	CR	Skin: white spots Neuro: Epilepsy, tubers, SEN, DD
8	f	5.1	prenatal	NA	sporadic	CR	SEGA	Skin: white spots Neuro: Epilepsy, tubers, SEN, white matter lesions, DD Other: retinal hamartomas
9	m	4	prenatal	*TSC2*	sporadic	CR	CR / SEGA	Skin: white spots Neuro: Epilepsy, tubers, SEN, migration lines, DD
10	m	4.1	30	NA	sporadic	CR	Epilepsy	Skin: white spots Neuro: tubers, SEGA, aberrant gyrification Other: renal angiomyolipoma
11	f	1.7	30	*TSC2*	sporadic	CR	CR	Neuro: Epilepsy, tubers, SEN, DD
12	m	2.1	0	*TSC2*	sporadic	SEGA	SEGA	Neuro: Epilepsy, tubers, SEN, white matter lesions, DD Cardiac: CR
13	m	2.6	15	*TSC2*	sporadic	CR	CR	Skin: white spots Neuro: tubers, SEN, DD
14	m	3.3	prenatal	*TSC 2*	sporadic	CR	CR	Skin: white spots, angiofibromas Neuro: Epilepsy, SEN, DD
15	m	0.9	prenatal	*TSC1*	sporadic	CR	CR	None
16	m	0.3	90	*TSC2*	sporadic	Epilepsy	Epilepsy	Neuro: tubers, SEN, DD Cardiac: CR Other: retinal hamartomas, renal angiomyolipoma
17	m	2.4	30	*None*	sporadic	CR	Epilepsy	Skin: white spots Neuro: tubers, SEN, DD

*CR* cardiac rhabdomyoma, *DD* developmental delay, *NA* not available, *SEGA* subependymal giant cell astrocytoma, *SEN* subependymal nodule

Genetic analysis was carried out in 12 of the 17 cases. One child carried a mutation in *TSC1* and 10 patients in *TSC2*. No mutation could be identified in patient #17. No genetic information was available in the remaining 5 cases. TSC occurred sporadically in 15 patients while family history was positive in 2 patients.

Three patients were born pre-term due to cardiac complications (patients #9, #11 and #13). All other patients were born at term with a normal birth weight and normal APGAR scores. Dermatologic manifestations were present in 11 cases, including white spots, facial angiofibromas, periungual fibromas, and a shagreen patch. Retinal hamartomas were present in 3 individuals. Two patients developed renal angiomyolipomas. Another patient showed an atypical manifestation with hemihypertrophy, congenital focal lymphedema of the leg, lumbar scoliosis and vascular anomalies including aneurysms and renal artery stenosis. Overall 14/17 patients (82.4%) suffered from epileptic seizures. Seizure types included focal, generalized and atonic seizures, and in one case infection-related status epilepticus with Todd’s palsy. IS developed in 7 cases (41.2%) with a median age at onset of 5.5 months (range 1-7 months). Neuroimaging revealed tubers in 14/17 patients (82.4%), subependymal nodules (SEN) in 12 (70.6%), and SEGA in 7 patients (41.2%). Neuroimaging was normal in 2 cases (11.8%). Initial neuropsychological assessment was normal in 5 of 14 tested children (35.7%). Nine patients (64.3%) showed various degrees of developmental delay. In 3 cases, no data concerning neurologic development were available.

**Fig. 1:**
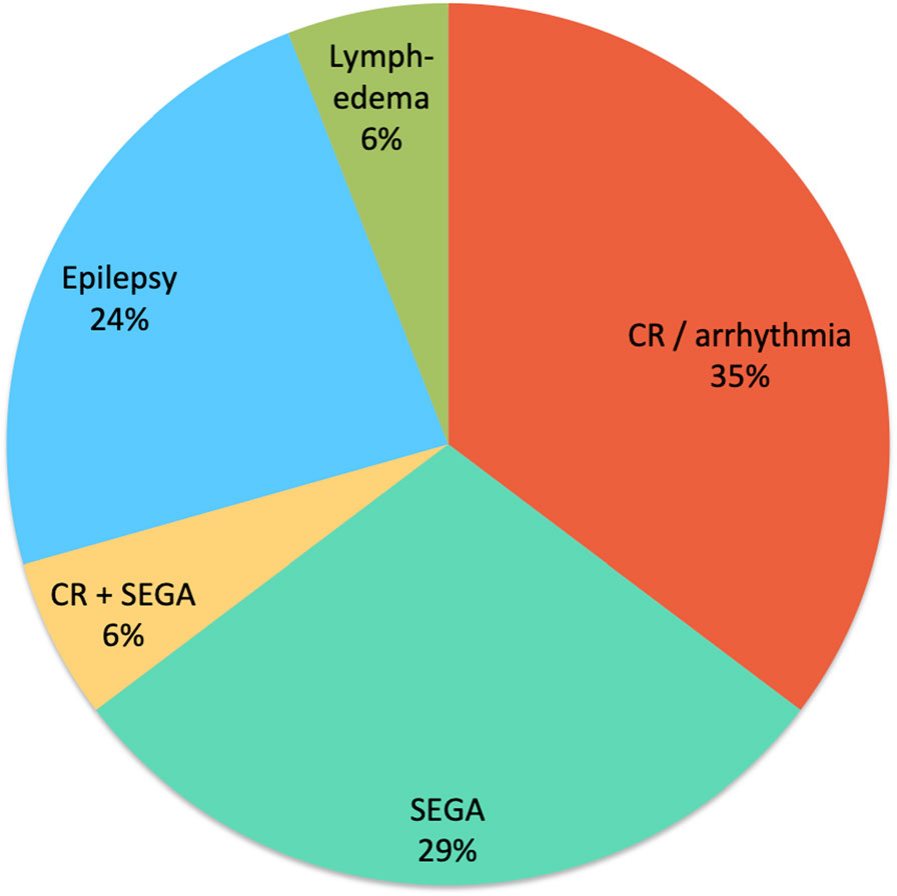
Reasons for initiation of mTOR inhibitor treatment. *CR* cardiac rhabdomyoma. *SEGA* subependymal giant cell astrocytoma.

Reasons for initiation of mTOR inhibitor treatment were symptomatic cardiac rhabdomyomas and arrhythmia in 6 (35.3%), SEGA in 5 (29.4%), the combination of cardiac rhabdomyoma and SEGA in 1 (5.9%), refractory epilepsy in 4 cases (23.5%) and congenital focal lymphedema in 1 case (5.9%) (Table 1**,** Fig. 1).

In all subjects everolimus (Certican™, Votubia™) was used as mTOR inhibitor. Everolimus was administered orally in all cases. Therapy was conducted with a median age at initiation of 5 months (range 0–19 months).

### Safety profile of everolimus therapy

Data on the safety profile of everolimus therapy are summarized in Table 2. Dosing regimens and absolute dosages varied significantly between centers. In general, newborns up to 3 months received everolimus in doses ranging from 0.05–0.3 mg daily, while infants older than 5 months received higher doses ranging from 1 to 5 mg daily.

**Table 2:** Safety profile of mTOR inhibitor therapy

Patient #	Age at start of treatment	Dosages	Comedications	AE	Vaccination status before therapy	Vaccination status during therapy
1	19 months	2.5 mg once daily	VGB, MAD	Recurrent URTI / LRTI Increase of cholesterol / TG	Complete	NA
2	14 months	2.5 mg 3 times per week	VGB, STM, VPA	None	Complete	No live vaccines
3	15 months	2.5 mg once daily	LTG, VGB	Transient stomatitis	Complete	NA
4	17 months	2 mg once daily	LEV, VPA	Transient stomatitis	Complete	NA
5	12 months	3 mg once daily	OXC	None	NA	No live vaccines
6	14 days	0.3 mg two times per week	flecainide, propranolol	None	Incomplete	NA
7	5 months	1 mg daily	ACTH, OXC, VGB, TMP	Transient anemia	No vaccinations	NA
8	14 months	2.5 mg daily	different AEDs, TMP	Recurrent URTI / LRTI Increase of cholesterol / TG	Complete	NA
9	7 days	NA	TPM, VGB, VPA, propanolol, melatonin	Recurrent infections Reduction of phosphate Increase of cholinesterase Increase of TG Increase of LDH	NA	Vaccinations under therapy
10	2 months	NA	LEV, VGB, sotalol	Recurrent infections	NA	Vaccinations during 3 months pause of therapy
11	7 days	0.05 mg every other day	VPA, VGB, TPM, propranolol, propafenone	Worsening of infantile acne Increase of phosphate Increase of cholinesterase	NA	Vaccinations under therapy
12	1 month	0.03 mg/m^2^twice per day	LEV, VGB	Transient neutropenia UTI	NA	NA
13	3 months	0.1 mg twice per day	VGB, flecainide, metoprolol, amiodarone, metildigoxin	Transient neutropenia Recurrent URTI	NA	NA
14	2 days	1.5-2 mg/m^2^ daily	VGB, LEV, digoxin, TMP/SMX, nystatin	Increase of cholesterol / TG Transient lymphopenia	NA	No live vaccines
15	2 days	0.25 mg daily	TMP/SMX, nystatin	None	NA	No live vaccines
16	7 months	0.5 mg twice per day	PB, VGB	Recurrent infections	NA	No live vaccines
17	10 months	5 mg daily	OXC, VGB, KD	None	NA	No live vaccines

*ACTH* adrenocorticotropic hormone, *AE* adverse event, *CR* cardiac rhabdomyoma, *DD* developmental delay, *KD* ketogenic diet, *LDH* lactate dehydrogenase, *LEV* levetiracetam, *LRTI* lower respiratory tract infection, *LTG* lamotrigine, *MAD* modified Atkins diet, *NA* not available, *OXC* oxcarbazepine, *PB* phenobarbital, *SEGA* subependymal giant cell astrocytoma, *SEN* subependymal nodule, *SMX* sulfamethoxazole, *STM* sulthiame, *TG* triglycerides, *TMP* trimethoprim, *TPM* topiramate, *URTI* upper respiratory tract infection, *UTI* urinary tract infection, *VGB* vigabatrin, *VPA* valproic acid

**Fig. 2:**
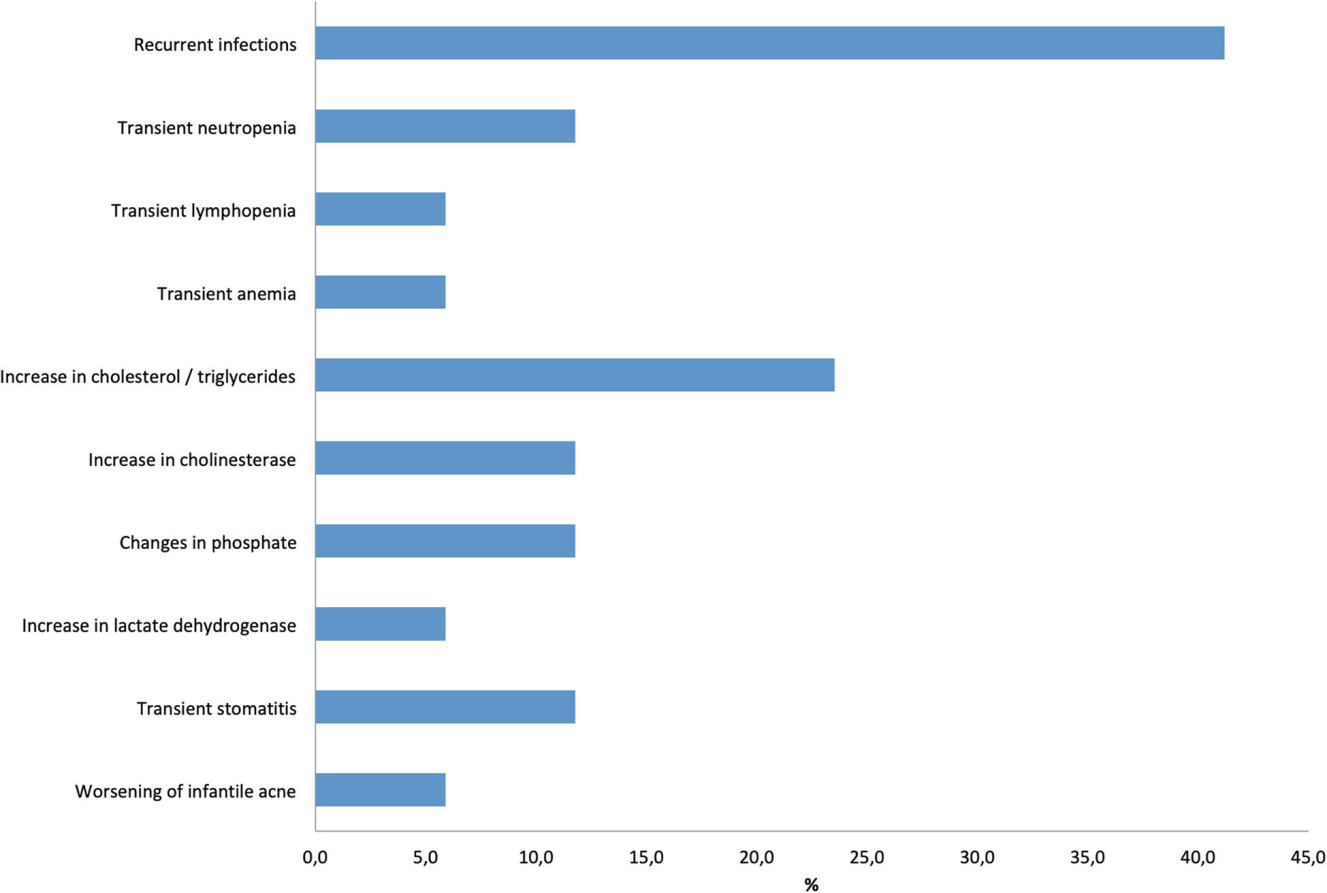
Reported adverse events during everolimus treatment

Adverse events were classified according to the *Common Terminology Criteria of Adverse Events* (CTCAE, Version 5.0 [14]). Grade 1–2 adverse events occurred in 12 cases with a median of 1 adverse event (range 0–5) per individual and included mild transient stomatitis (2 cases), worsening of infantile acne (1 case), increase of serum cholesterol and triglycerides (4 cases), changes in serum phosphate levels (2 cases), increase of cholinesterase (2 cases), increase of serum lactate dehydrogenase (1 case), transient neutropenia (2 cases), transient anemia (1 case), transient lymphopenia (1 case) and recurrent infections (7 cases) (Fig. 2). No grade 3–4 adverse events were reported. Treatment is currently ongoing in 13 of 17 patients. Everolimus treatment was discontinued due to rapid shrinkage of a cardiac rhabdomyoma after 19 days of treatment without rebound tumor growth within a 5 month follow-up period in patient #14 [13], because of stable disease after significant reduction of a SEGA in patient #12, presurgically before removal of an epileptogenic tuber in patient #16, and due to lack of improvement of seizures in patient #17.

Comedications during everolimus therapy included vigabatrin, valproate, levetiracetam, oxcarbazepine, topiramate, lamotrigine, sultiame, phenobarbital, ACTH, propranolol, flecainide, metoprolol, amiodarone, metildigoxin, propafenone, sotalol, trimethoprim, ketogenic diet and modified Atkins diet. In one girl (patient #7) a decrease in trough levels of oxcarbazepine was observed after initiation of everolimus. No other relevant interactions with everolimus treatment were reported.

Routine check-ups were performed in 2 monthly to yearly intervals and included routine laboratory work-up with complete blood count, liver and kidney studies, serum triglycerides and cholesterol as well as drug trough levels, EEG studies and cardiac and neurologic imaging.

Vaccination status and social integration of children before and during everolimus therapy were assessed where data were available (Table 2). In 5/17 children vaccination status was completed in accordance to official recommendations before initiation of everolimus treatment. During treatment live vaccines were avoided in 6 patients. In one child vaccinations were performed during a treatment pause of 3 months (patient #10). In two patients, who were treated with everolimus in the neonatal period, vaccinations including live vaccines were conducted according to official recommendations despite everolimus treatment (patient #9 and #11). No vaccination related adverse events were reported.

### Changes of cardiac manifestations and SEGA size under everolimus therapy

Sixteen of 17 patients (94.1%) showed cardiac rhabdomyomas at birth. In 7 of these 16 cases (43.8%) everolimus was initiated due to obstruction of cardiac outflow or cardiac arrhythmias. In all of these 7 cases, and in one additional case treated for SEGA (patient #12), rhabdomyomas decreased in size under everolimus therapy (Table 3). In one case (patient #13) cardiac arrhythmias had been refractory to treatment with flecainide, metoprolol, amiodarone and metildigoxin. Initiation of everolimus therapy at 3 months of age led to rapid decrease of associated rhabdomyoma size and therapeutic control of cardiac arrhythmias within 1 month. Discontinuation of treatment in the presence of stable disease at 4 months of age however caused regrowth of the cardiac rhabdomyoma and recurrence of the arrhythmias warranting restart of everolimus therapy with again rapid normalization of the cardiac rhythm pattern. A second attempt to withdraw everolimus during the second year of life again led to recurrence of arrhythmias, barely controllable under quadruple anti-arrhythmic therapy. Addition of everolimus again alleviated disease severity.

**Table 3:** Effect of mTOR inhibitor therapy on TSC-related manifestations and neurodevelopment

Patient #	Reason for therapy	Effect of therapy	Development before therapy	Development at follow-up
1	SEGA	Decrease of SEGA	Normal	DD (not specified)
2	Congenital focal lymphedema	Regression of congenital focal lymphedema	DD (global)	DD (not specified) *K-ABC, WPPSI-III*
3	SEGA	Decrease of SEGA	DD (motor)	Normal *SON-R*
4	SEGA	Decrease of SEGA	DD (not specified)	DD (global) *MFED*
5	Epilepsy	Decrease of seizure frequency	DD (not specified)	NA
6	CR	Decrease of CR	Normal	Normal
7	CR	Decrease of CR	Normal	DD (speech) *MFED*
8	SEGA	No effect	DD (not specified)	DD (global) *Bayley II*
9	CR / SEGA	Decrease of CR / SEGA	NA	DD (global)
10	Epilepsy	Ongoing seizures	NA	Normal
11	CR / cardiac arrhythmia	Decrease of CR	NA	DD (global)
12	SEGA	Decrease of CR / SEGA	Normal	DD (global) *Bayley II*
13	CR / cardiac arrhythmia	Decrease of CR	DD (motor)	DD (motor) *Bayley II, VABS II, CBCL 1½-5, ELFRA-2*
14	CR	Decrease of CR	DD (global)	DD (motor)
15	CR	Decrease of CR	Normal	NA
16	Epilepsy	Decrease of seizure frequency	DD (not specified)	DD (global) *Bayley II, GMDS*
17	Epilepsy	Ongoing seizures	DD (motor)	DD (not specified)

*CBCL* Child Behavior Checklist, *CR* cardiac rhabdomyoma, *DD* developmental delay, *ELFRA* Elternfragebogen für die Früherkennung von Risikokindern, *GMDS* Griffith Mental Developmental Scales, *K-ABC* Kaufman Assessment Battery for Children, *MFED* Münchener Funktionelle Entwicklungsdiagnostik, *NA* not available, *SEGA* subependymal giant cell astrocytoma, *SON-R* Snijders-Oomen non-verbal intelligence test, *VABS* Vineland Adaptive Behavior Scale, *WPPSI* Wechsler Preschool and Primary Scale of Intelligence

Seven of 17 patients (41.2%) developed SEGA during the observation period. In 6/7 patients everolimus was started due to growing or symptomatic SEGA. In 5 of these 6 patients (83.3%) SEGA size decreased significantly during therapy, while in 1 patient (patient #8) no effect was observed (Table 3**,** Fig. 4). Among the patients treated for SEGA, 2 patients (patient #9 and #12) were treated within the first month of life. A significant reduction of SEGA size was documented on short time follow-up MRI after 2 and 3 months of treatment respectively.

### Epilepsy and neurodevelopment under everolimus therapy

Epilepsy was diagnosed in 14/17 patients (82.4%) during their first 2 years of life. In 4/17 (23.5%) everolimus treatment was started for treatment-resistant epilepsy (Table 3). One patient (patient #5) showed marked improvement and became almost free of seizures on combination therapy with everolimus and oxcarbazepine. The second patient (patient #10) was highly refractory to anticonvulsants including levetiracetam, oxcarbazepine, sultiame, vigabatrin, pregabalin, pheytoin, pyridoxine and corticosteroids. He had epilepsy surgery with removal of a cortical tuber and was started on everolimus. However, despite a combination of vigabatrin, levetiracetam and everolimus, he still suffers from daily seizures. Similarly, the third patient (patient #17), experiencing refractory seizures under levetiracetam, oxcarbazepine, vigabatrin, ketogenic diet and everolimus, underwent epilepsy surgery, which only led to short-term seizure freedom. The fourth patient (patient #16), by contrast, previously experiencing refractory focal seizures under levetiracetam, vigabatrin, phenobarbital and ketogenic diet, showed 50% reduction of seizure frequency after start of everolimus and was seizure free under temporary toxic everolimus trough levels of 97.4 ng/ml. On reduction of everolimus to non-toxic levels of 14-18 ng/ml seizures recurred. Finally the patient underwent epilepsy surgery and is currently free of seizures.

Seven of 17 patients (41.2%) developed IS during the observation period (Table 4**).** IS occurred at a median age of 5.5 months (range 1–7 months). Primary treatments included ACTH (*n* = 1) or vigabatrin (*n* = 7). In 5 cases cessation of spasms occurred after a median duration of 3 months. No data were available for the remaining 2 cases. Eight of 17 patients received everolimus treatment within the neonatal period (first 3 months of life) prior to the vulnerable period for IS. The remaining 9 patients were started on everolimus treatment after 5 months of age. IS occurred in both groups. Three of 8 patients (37.5%) in the neonatal group developed IS while 4/9 patients (44.4%) in the group of older children were affected. Neurodevelopment was abnormal in both groups on follow-up (Table 4).

**Table 4:** Effect of mTOR inhibitor therapy on IS and neurodevelopment

Patient #	Age at start of mTOR inhibitor	AAO of IS	Previous treatment	Time to cessation of IS	Development prior to start of mTOR inhibition	Development during follow-up
1	19 months	7 months	VGB	16 months	Normal	DD (not specified)
2	14 months	5 months	VGB	3 months	DD (global)	DD (not specified)
3	15 months	6 months	VGB	NA	DD (motor)	Normal
7	5 months	NA	ACTH, VGB	1 month	Normal	DD (speech)
9	7 days	7 months	VGB	4 months	NA	DD (global)
11	7 days	1 month	VGB	NA	NA	DD (global)
12	1 day	5 months	VGB	1 month	NA	DD (global)

*AAO* age at onset, *IS* infantile spasms

Neurodevelopment prior to initiation of everolimus treatment was evaluated in 14/17 children (Table 3). Due to the lack of valid scoring systems in very young children, developmental assessment was mostly carried out by pediatricians and rated based on clinical exam and the achievement of age-expected psychomotor milestones. While in 5 cases (35.7%) development was reported as normal (patients #1, #6, #7, #12 and #15), 9 patients (64.3%) showed various degrees of developmental delay. Reported findings included delays in the acquisition of a social smile, delayed achievement of speech or motor milestones (mostly unsupported sitting) and the presence of muscular hypotonia or focal neurologic deficits. On follow-up, 15 patients were tested (Table 3). Data on structural assessments were available in 8 cases and included Bayley II, K-ABC (*Kaufman Assessment Battery for Children*), WPPSI-III (*Wechsler Preschool and Primary Scale of Intelligence*), SON-R (*Snijders-Oomen non-verbal intelligence test*), MFED (*Münchener Funktionelle Entwicklungsdiagnostik*), VABS II (*Vineland Adaptive Behavior Scale*), CBCL 1½-5 (*Child Behavior Checklist*), ELFRA-2 (*Elternfragebogen für die Früherkennung von Risikokindern*) and GMDS (*Griffith Mental Developmental Scales*) testing. Three patients (20%) had normal results (patients #3, #6 and 10), while 12 (80%) showed a wide spectrum of developmental delay ranging from mild motor or speech delay to global developmental delay. Interestingly, one patient (patient #2), reported with severe global developmental delay on initial testing, showed significant neurodevelopmental improvement after the start of everolimus at the age of 14 months.

## Discussion

We collected data from 17 TSC patients from 12 referring centers in Germany. All of these children received everolimus therapy in the first 2 years of life. Treatment was mainly initiated for symptomatic cardiac rhabdomyomas (35.3%), SEGA (29.4%) or the combination of both (5.9%). Other reasons for mTOR inhibition were refractory epilepsy (23.5%) and one rare case of congenital focal lymphedema of the leg (5.9%).

### Safety profile

Everolimus was the only mTOR inhibitor used in our study. Everolimus therapy was overall well tolerated (Table 2). Grade 1–2 adverse events occurred in 70.6% of patients and included dermatologic manifestations (mild transient stomatitis, worsening of infantile acne), subclinical laboratory changes (increase of cholesterol and triglycerides, changes in phosphate levels, increase of cholinesterase and lactate dehydrogenase, transient anemia and neutropenia) as well as uncomplicated infections. During winter and under high everolimus trough levels, one patient (patient #1) showed recurrent viral respiratory tract infections, repeatedly complicated by bacterial superinfection requiring antibiotic therapy. No grade 3–4 adverse events were reported. Routine laboratory checks, in most cases monthly blood tests, proved as helpful.

Treatment is currently ongoing in 76.5% and in the remaining 23.5% was discontinued due to stable disease with reduced SEGA volume in one case (patient #12), reduction in cardiac rhabdomyoma size in one child (patient #14 [13]), before epilepsy surgery in another patient (patient #16) and due to refractory seizures despite treatment in the last patient (patient #17).

In most patients vaccinations with live-attenuated vaccines were not performed or in one case only in the context of a treatment pause of 3 months (patients #10). In 2 cases vaccinations including live-attenuated vaccinations were performed despite everolimus treatment and without adverse events. No data regarding immune response during vaccinations with everolimus were available. However, in the elder population everolimus treatment has been previously reported as safe and helpful to induce an immune response [15].

### Dosing

Dosing regimens and absolute doses of everolimus varied significantly and ranged from 0.05–0.3 mg daily in neonates to 1–5 mg daily in children older than 5 months. No specific dosing recommendations for everolimus are available for neonates so far, however, previous studies have shown that the primary elimination pathway of everolimus, i.e. the CYP3A4 pathway, is extremely weak or absent in neonates, highlighting the need for cautious use and repetitive drug monitoring [16, 17]. Along these lines two patients in our study showed toxic everolimus trough levels of around 100 ng/ml after an initial dose of 0.4–0.45 mg (=1.5-2 mg/m^2^) [13] and under a dose of 2 mg twice daily (=0,4 mg/kg) (patient #16) respectively.

We searched the literature for reported cases of everolimus treatment in neonates (Table 5). All reported neonates were treated for cardiac rhabdomyomas. The median dose was 0.64 mg/m^2^/day (range 0.12–1.0 mg/m^2^/day), targeting median trough levels of 10.2 ng/ml (range 4.5–13.7 ng/ml). This dose is significantly lower than recommended doses in children above 3 years of age (3.0 mg/m^2^/day) for SEGA treatment [18, 19].

**Table 5:** Summary of previous reports on mTOR inhibitor dosing in neonates

mTOR inhibitor	AAO of mTOR inhibitor	Dosing regimen	Dose [mg/m2/d]	Trough levels [ng/ml]	Duration	AE	Prophylaxis	Reference
EVE	NA	0.25 mg twice per day 2 days per week	NA	5–15	2.5 months	Increase of TG Self-limiting diarrhea Decreased CD/CD8 ratio lymphopenia	TMP/SMX	(Demir et al. 2012)
Sirolimus	10 days	0.4 mg daily	NA	NA	24 days	Increase of TG	TMP/SMX	(Breathnach et al. 2014)
EVE	20 days	0.1 mg daily	0.64	11	34 days	Transient hypokalemia	NA	(Mohamed et al. 2014)
EVE	2 days	0.25 mg twice per day 2 days per week	NA	3.6–7.8	3 months	NA	NA	(Dogan et al. 2015)
EVE	4 days	0.1 mg daily	0.64	11.5	42 days	NA	NA	(Goyer et al. 2015)
EVE	9 days	0.1 mg daily	0.64	10.2	NA	NA	NA	
EVE	21 days	NA	3	4–5	NA	Hyponatremia	antibiotic prophylaxis	(Mlczoch et al. 2015)
EVE	NA	0.25 mg twice per day 2 days per week	NA	NA	4 weeks	NA	NA	(Oztunc et al. 2015)
EVE	2 days	0.15–0.2 mg daily	1	10–15	19 days	Toxic levels of 108 ng/ml under initial dose of 0.4–0.45 mg (=1.5-2 mg/m^2^)	NA	(Wagner et al. 2015)
EVE	7 days	0.25 mg daily	1	NA	10 weeks	Mild mucositis Increase of TG	NA	(Colaneri et al. 2016)
EVE	NA	0.25 mg twice per day 2 days per week	NA	5–10	NA	NA	NA	(Emir et al. 2017)
EVE	NA	0.5 mg daily	NA	NA	NA	NA	NA	(Hoshal et al. 2016)
EVE	20 days	0.1 mg daily	NA	13.7	34 days	Suspected infection	NA	(Aw et al. 2017)
EVE	4 days	NA	NA	11	42 days	None	NA	
EVE	9 days	0.1 mg daily	NA	10.2	NA	Oral ulcers	NA	
EVE	1 day	0.1 mg daily	NA	10.2	36 days	None	NA	
EVE	NA	0.03125 mg daily	0.12	3–7	2 weeks	Adenovirus pneumonia High trough levels of 20 ng/ml under 0.125 mg (0.0558 mg/m^2^)	NA	(Chang et al. 2017)
EVE	NA	NA	0.35	1.81	NA	NA	NA	
EVE	NA	0.125 mg daily	0.51	NA	Na	NA	NA	

*AAO* age at onset, *AE* adverse events, *EVE* everolimus, *NA* not available, *TG* triglycerides, *TMP/SMX* trimethoprim/sulfamethoxazole

### Outcomes

**Fig. 3:**
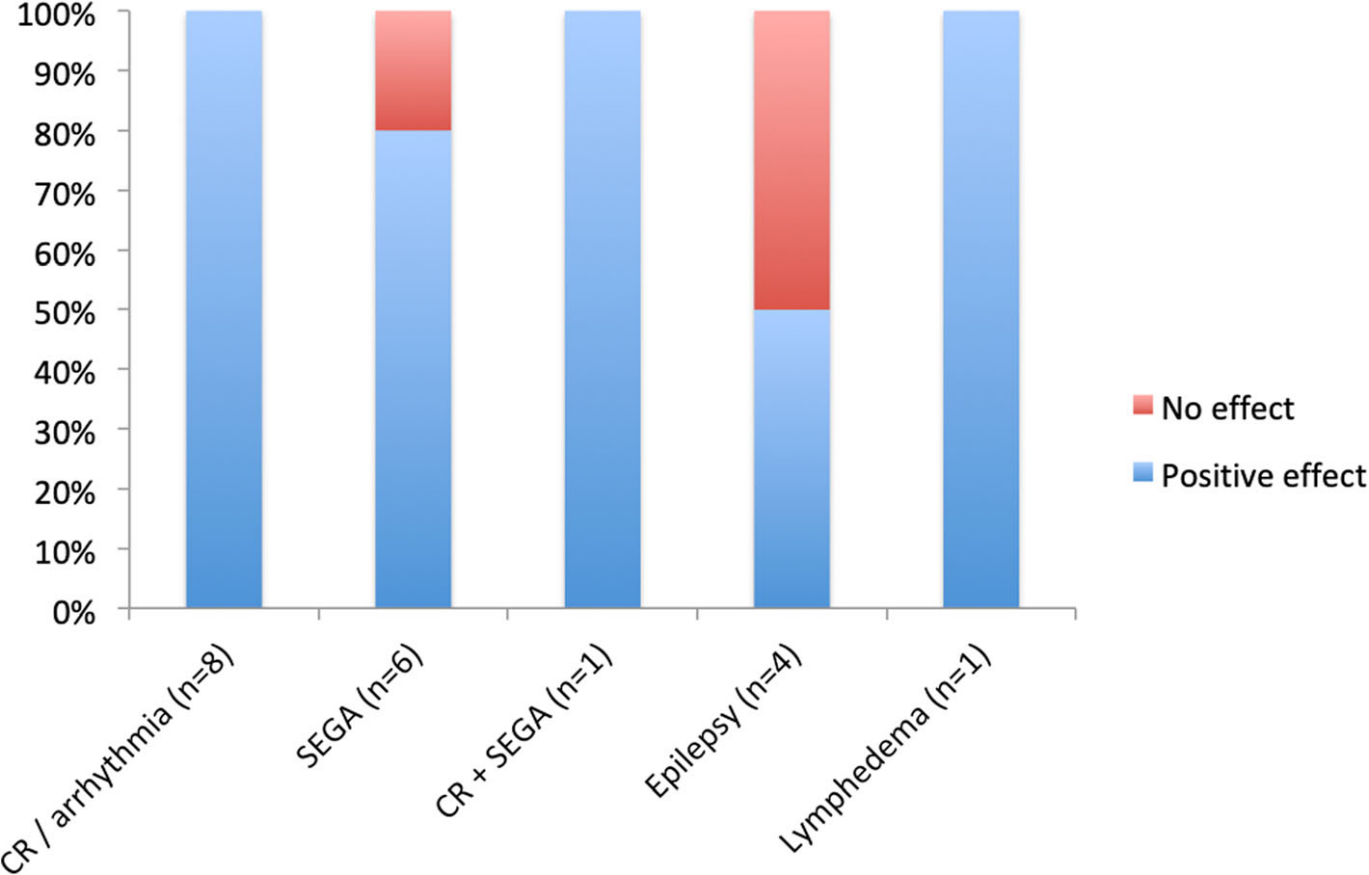
Effects of everolimus treatment. *CR* cardiac rhabdomyoma. *SEGA* subependymal giant cell astrocytoma.

Therapeutic benefits following everolimus treatment were present in more than 80% of patients (Fig. 3) and included decrease of cardiac rhabdomyoma size and reduction of arrhythmias (8/8), decrease of SEGA size (5/6), regression of congenital focal lymphedema (1/1) and reduction of seizure frequency (2/4). Neither patient #8, treated for SEGA, nor patients #10 and #17, treated for highly refractory epilepsy, showed improvement under everolimus.

### Cardiac manifestations

Cardiac rhabdomyoma size rapidly decreased in all patients treated with everolimus. Of special interest is the case of patient #13. In this child discontinuation of everolimus therapy after sustained reduction of a cardiac rhabdomyoma repeatedly led to a regrowth and recurrence of potentially life-threatening arrhythmias warranting restart of everolimus, again resulting in prompt control of the arrhythmias that could not be achieved by anti-arrhythmic medication alone. This observation applies for beneficial effects of mTOR inhibition also on the excitability of electrically active cardiomyocytes in TSC-related arrhythmias comparable to its anticonvulsant effects in neurons. In 85.7% of patients treated for cardiac rhabdomyomas, everolimus was started within the first 3 months of life. These findings are in line with several reports and small case series in the literature similarly demonstrating relevant cardiac rhabdomyoma size reduction in neonates with high-risk for cardiac surgery, treated with everolimus [13, 20–31]. Our series confirms that neonatal everolimus treatment is overall well tolerated and beneficial for those infants even for prolonged treatment.

### Sega

**Fig. 4:**
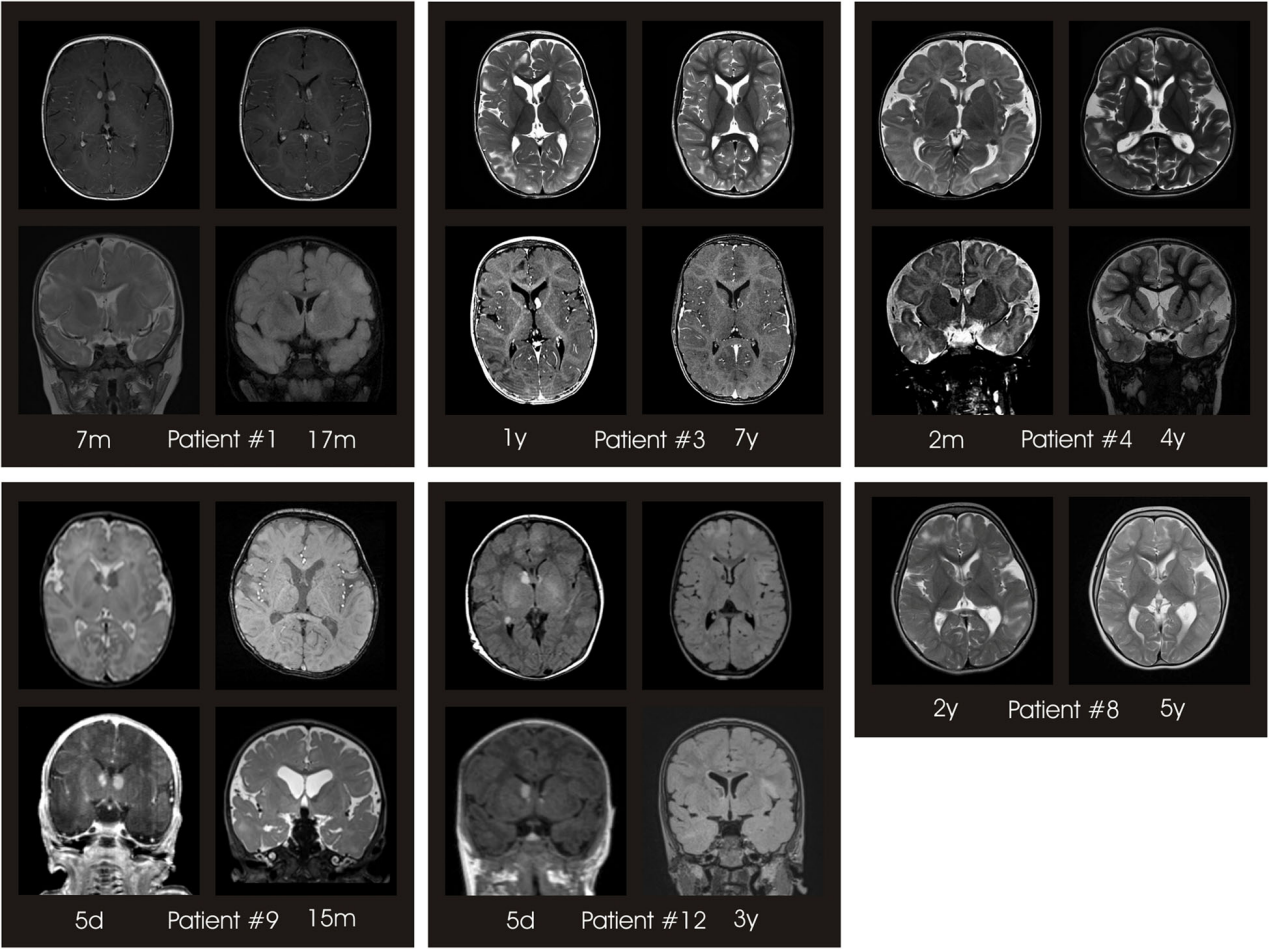
SEGA development under everolimus treatment. Axial and coronal T2-weighted and T1-weighted with gadolinium MRI sections of 6 patients treated with everolimus for SEGA. All patients were diagnosed with SEGA at risk for development of obstructive hydrocephalus at baseline *(left column)*. On follow-up in 5/6 patients marked reduction of SEGA volume was documented *(patient #1, 3, 4, 9, 12, right* ***column)***. In patient #8 SEGA size remained unchanged after initiation of everolimus treatment ***(right column)***

SEGA size reduced significantly in the majority (83.3%) of patients (Table 3). No patient required surgical removal of SEGA during the study period. Two patients with SEGA received everolimus in the neonatal period (patients # 9 and #12) with beneficial effects on tumor size (Fig. 4). Data on SEGA treatment in children below 2 years of age are still scarce. Kuki and colleagues successfully used everolimus in 5 children under the age of 12 months and reported rapid SEGA volume reduction of at least 50% in all treated patients within 6 months [32]. Everolimus is currently widely used and approved for the early treatment of SEGA. Our study provides further evidence for the safety and beneficial effects of everolimus therapy for TSC-related SEGA under the age of 2 years.

### Other manifestations

One of our patients presented with congenital focal lymphedema, a very rare presentation of TSC. The frequency of congenital lymphedema in TSC patients has been reported with 0.7% and only 11 patients have been reported in the literature so far [33–40]. In our patient everolimus treatment showed significant positive effects on tumor texture and mobility of the affected limb (case report under review).

### Epilepsy

Epilepsy is a prevalent feature of TSC and the mTOR pathway plays major roles in epileptogenesis [41]. Unlike classical anticonvulsive drugs targeting ion channels, synaptic receptors or modifying neurotransmitter release, everolimus directly modifies downstream mechanisms involved in TSC-related epileptogenesis. Thus, mTOR inhibition is a tempting approach for TSC-related refractory epilepsy, underlined by the results of the EXIST-3 trial that showed sustained reduction of seizure frequency following adjunctive everolimus therapy in TSC-related refractory epilepsy in patients above 2 years [5]. Our cohort is too small to draw definitive conclusions on the benefits of everolimus treatment in TSC-related epilepsy under 2 years of age. More than 80% of children in our cohort developed seizures. Four were treated with everolimus for refractory epilepsy. While two patients (patients #10 and #17) are still suffering from daily seizures despite the use of various antiepileptic regimens, including everolimus and epilepsy surgery, one patient is currently seizure free under combination therapy with everolimus and oxcarbazepine (patient #6). In the fourth patient (patient #16) seizure frequency reduced to 50% under everolimus therapy. Interestingly, trough levels within the toxic range led to temporary seizure freedom, suggesting dose-dependent efficacy of everolimus in this child. In our cohort everolimus was effective as adjunctive antiepileptic therapy in 2 of 4 children, corroborating the results of the EXIST-3 trial [5]. Still, further studies are needed to investigate the benefits of mTOR inhibition on TSC-related epilepsy in infancy and early childhood.

### Neurodevelopment and infantile spasms

Neurodevelopment prior to initiation of everolimus treatment was evaluated in 82.4% of children. While in 35.7% of these children development was reported as normal, 64.3% showed psychomotor delay. On follow-up, 88.2% of patients were evaluated. 20% of these had normal results and 80% of patients showed circumscribed to global developmental delay. Interestingly, one patient (patient #2) showed significant neurodevelopmental improvement after the start of everolimus at the age of 14 months.

Neurodevelopmental outcome in TSC is closely linked to IS [6, 7]. The literature on everolimus treatment in TSC-related IS is scarce and only anecdotal reports exist [10, 42]. Recently, Samueli and colleagues reported improved neurocognitive outcome in 3 of 4 children with TSC-related IS under everolimus treatment. The prevalence of IS in our cohort was 41.2%. Interestingly, IS occurred in 37.5% of patients treated with everolimus during the first 3 months of life, while 44.4% of patients with everolimus initiation after 5 months of age developed IS (Table 4). Our patient cohort is currently too small to draw solid conclusions about potential benefits of early everolimus therapy on the occurrence of TSC-related IS and on neurodevelopmental outcome. Further studies are needed to evaluate the prophylactic use of everolimus in the neonatal period regarding IS and neurodevelopment.

## Conclusions

We provide detailed evidence that everolimus treatment is safe and efficacious in TSC patients under the age of 2 years, mostly allowing improved long-term outcome. The treatment was overall well tolerated and adverse events were mild in the great majority of cases. Benefits were reported in most cases including reduction of cardiac rhabdomyomas, improvement of cardiac arrhythmia, shrinkage of SEGA size, regression of congenital focal lymphedema and reduction of seizure frequency.

Limitations of this study are a small cohort and the still short follow-up period with a median follow-up of 2.1 years (range 0.5–5.5 years) since start of everolimus treatment. Thus, no predictions on long-term safety under maintenance therapy can be made. However, so far no overt adverse events have been reported.

Further multicenter studies and registers with larger cohorts and longer follow-up periods will determine the long-term safety of early everolimus therapy in infancy and early childhood and the effects on TSC-related neurodevelopmental disorders.

## Methods

To identify children with TSC and mTOR inhibitor treatment, we contacted all TSC centers in Germany. Seventeen patients from 12 different TSC clinics were identified that fulfilled the inclusion criteria (patient with a definitive diagnosis of TSC according to the *2012 International TSC Consensus Conference* [12] and treatment with an mTOR inhibitor started before 2 years of age). Data were collected and rated by one primary investigator per site. All patients and their parents or legal guardians gave informed consent for individual treatment with mTOR inhibitors and sharing of clinical data.
